# Supplementary material for: Highly nitrogen doped carbon nanofibers with superior rate capability and cyclability for potassium ion batteries
Source: Nat Commun. 2018 Apr 30;9:1720. doi: 10.1038/s41467-018-04190-z (PMC5928078; doi:10.1038/s41467-018-04190-z)
Supplement: Supplementary file 1 — Supplementary Information [file 41467_2018_4190_MOESM1_ESM.pdf]

Supplementary Information for

Highly nitrogen doped carbon nanofibers with superior rate capability and cyclability for potassium ion batteries

Xu et al.

Supplementary Figures

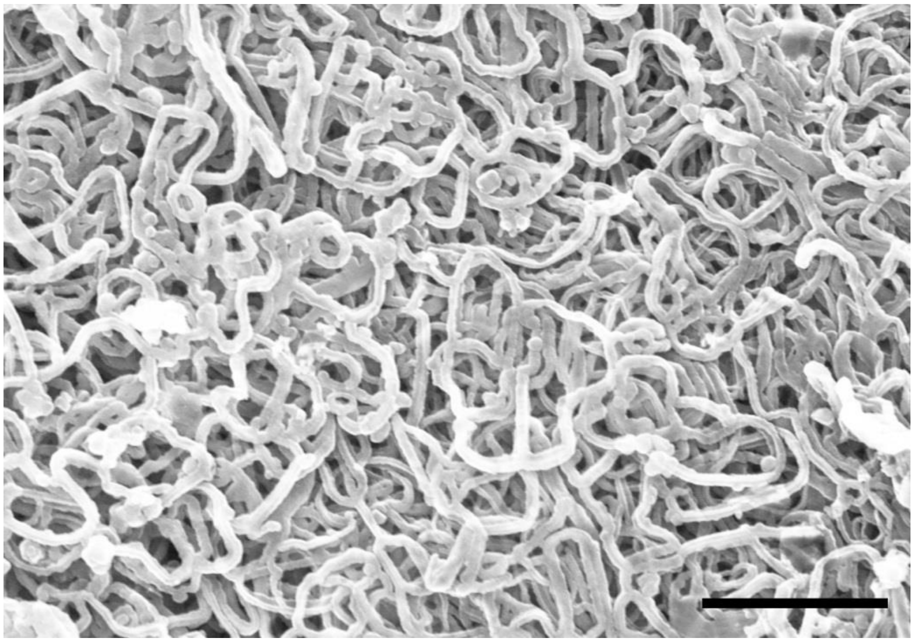

Supplementary Figure 1 | Morphology of the PPy precursor. SEM image of the PPy nanofiber precursor. Scale bar: 1 μm

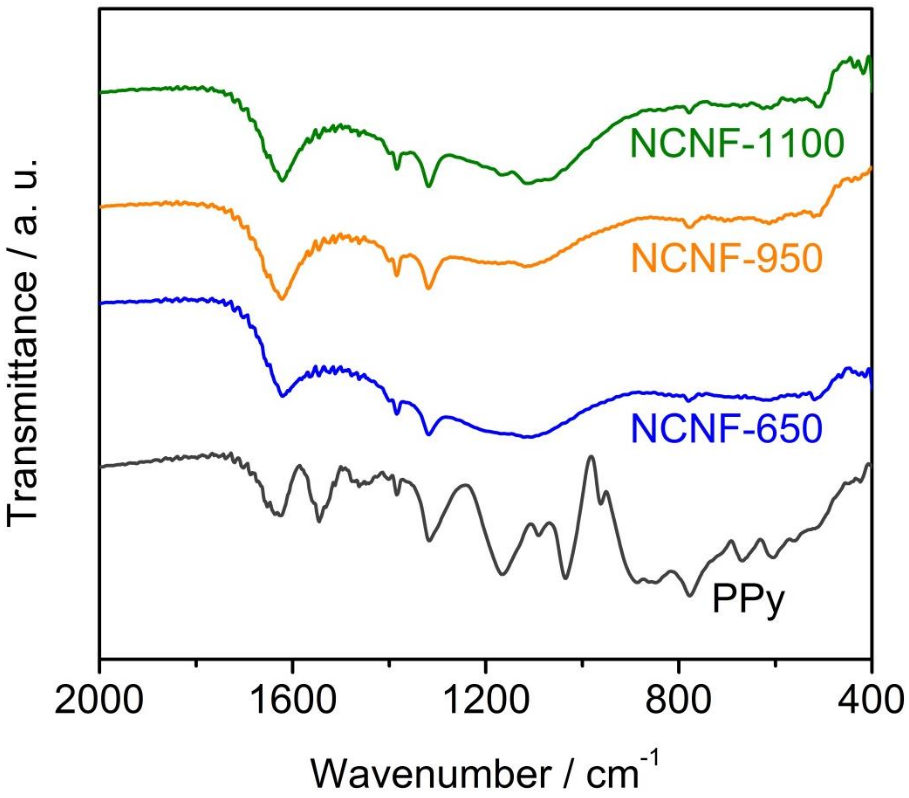

Supplementary Figure 2 | Infrared information of the PPy precursor and NCNFs. FTIR spectra of the PPy precursor and NCNFs.

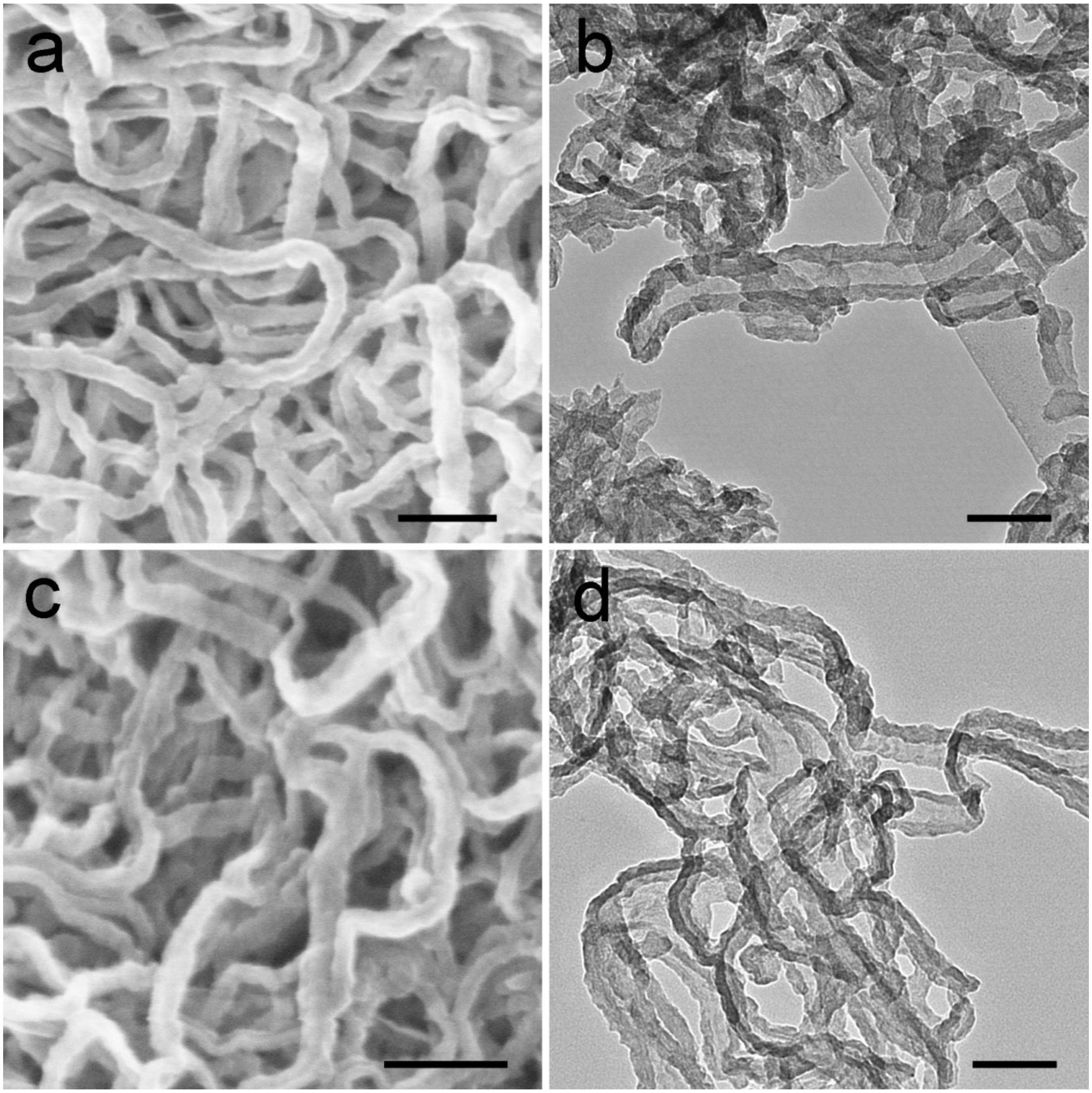

Supplementary Figure 3 | Morphology of NCNF-950 and NCNF-1100. SEM (a,c) and TEM images (b,d) of NCNF–950 (a,b) and NCNF–1100 (c,d). Scale bars: 200 nm (a and c); 100 nm (b and d).

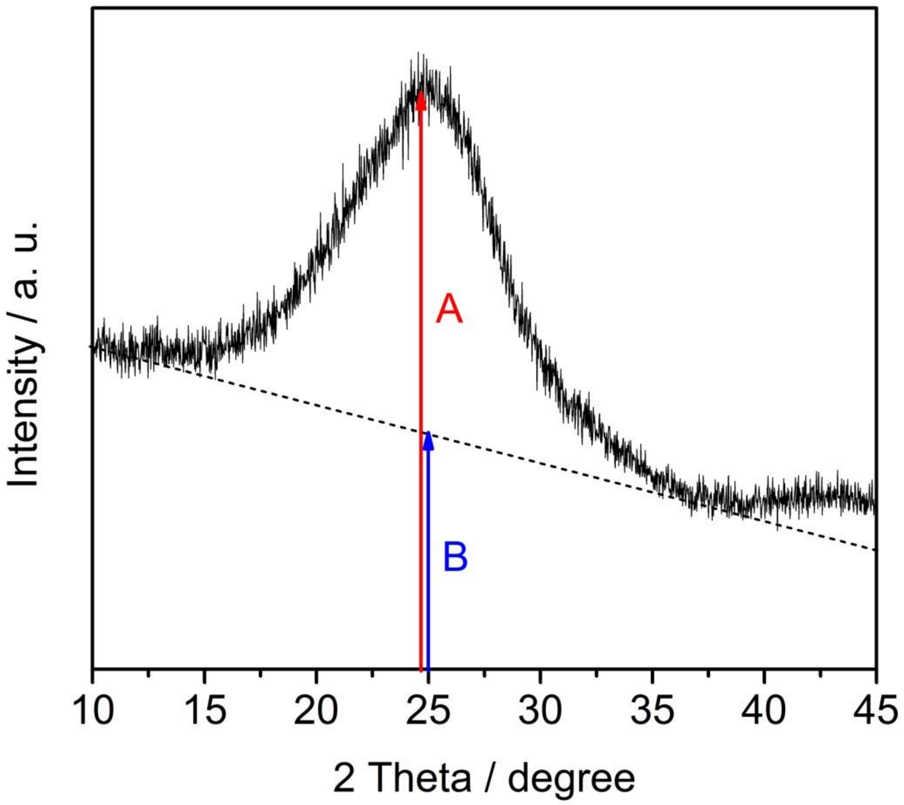

Supplementary Figure 4 | Calculation of the *R* value. Illustration of the calculation of the empirical *R* value according to the study of Dahn et al. The XRD pattern of NCNF-650 is used as an example.

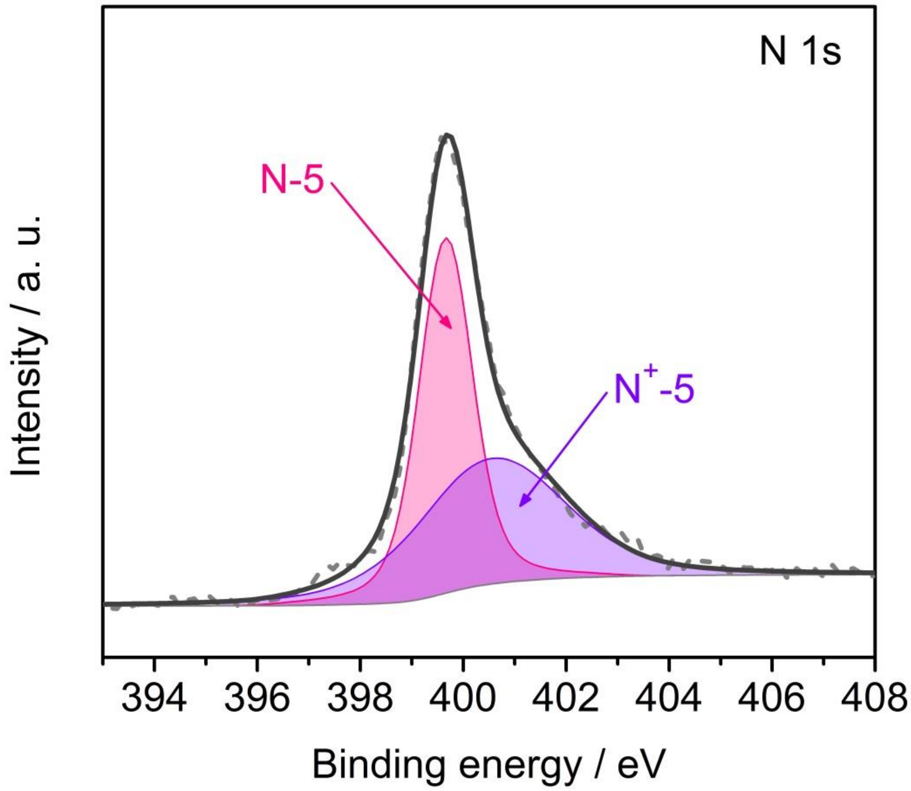

Supplementary Figure 5 | Surface chemistry of the PPy precursor. High-resolution N 1s core level XPS spectrum of the PPy precursor.

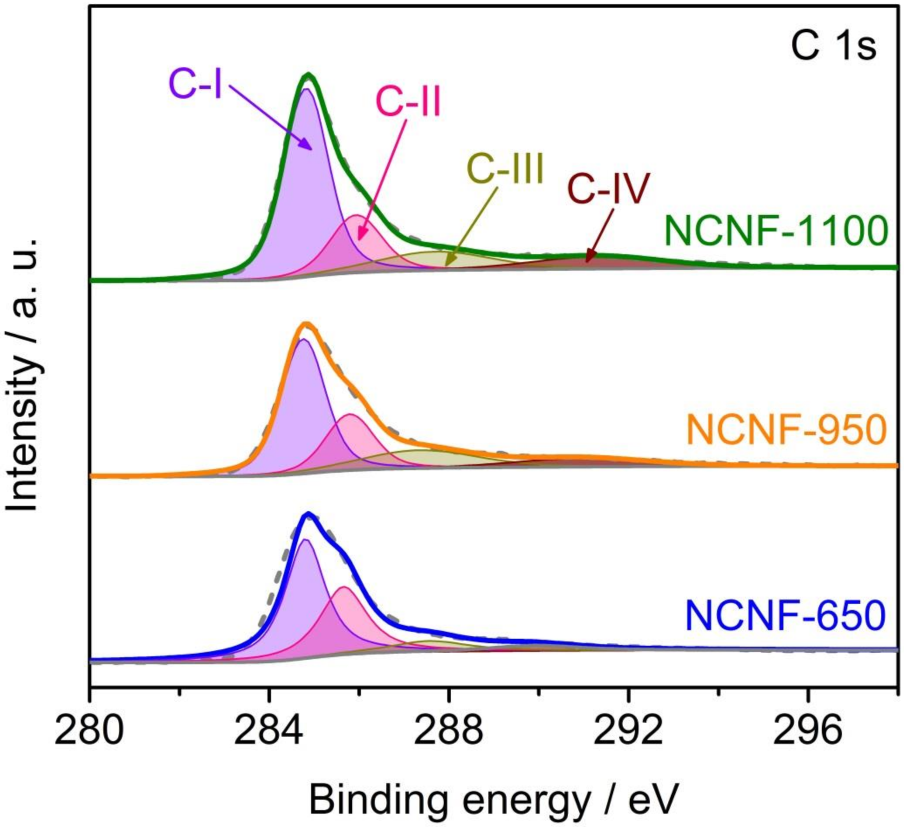

Supplementary Figure 6 | Surface chemistry of NCNFs. High-resolution C 1s core level XPS spectra of NCNFs.

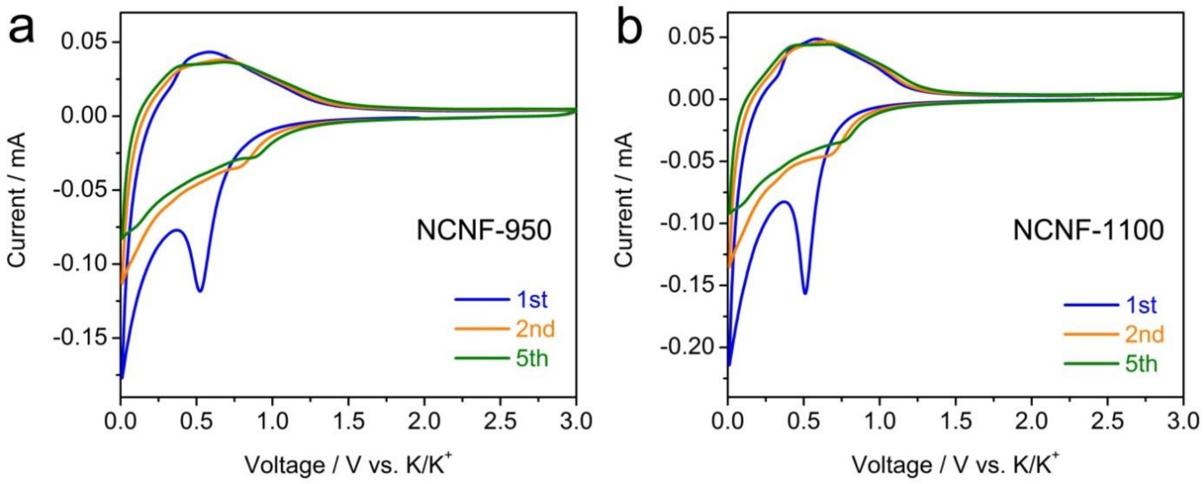

Supplementary Figure 7 | CV characterization of NCNF-950 and NCNF-1100. CV curves of NCNF-950 (a) and NCNF-1100 (b) tested at scan rate of 0.1 mV s^{-1} in a voltage window of 0.01–3.0 V.

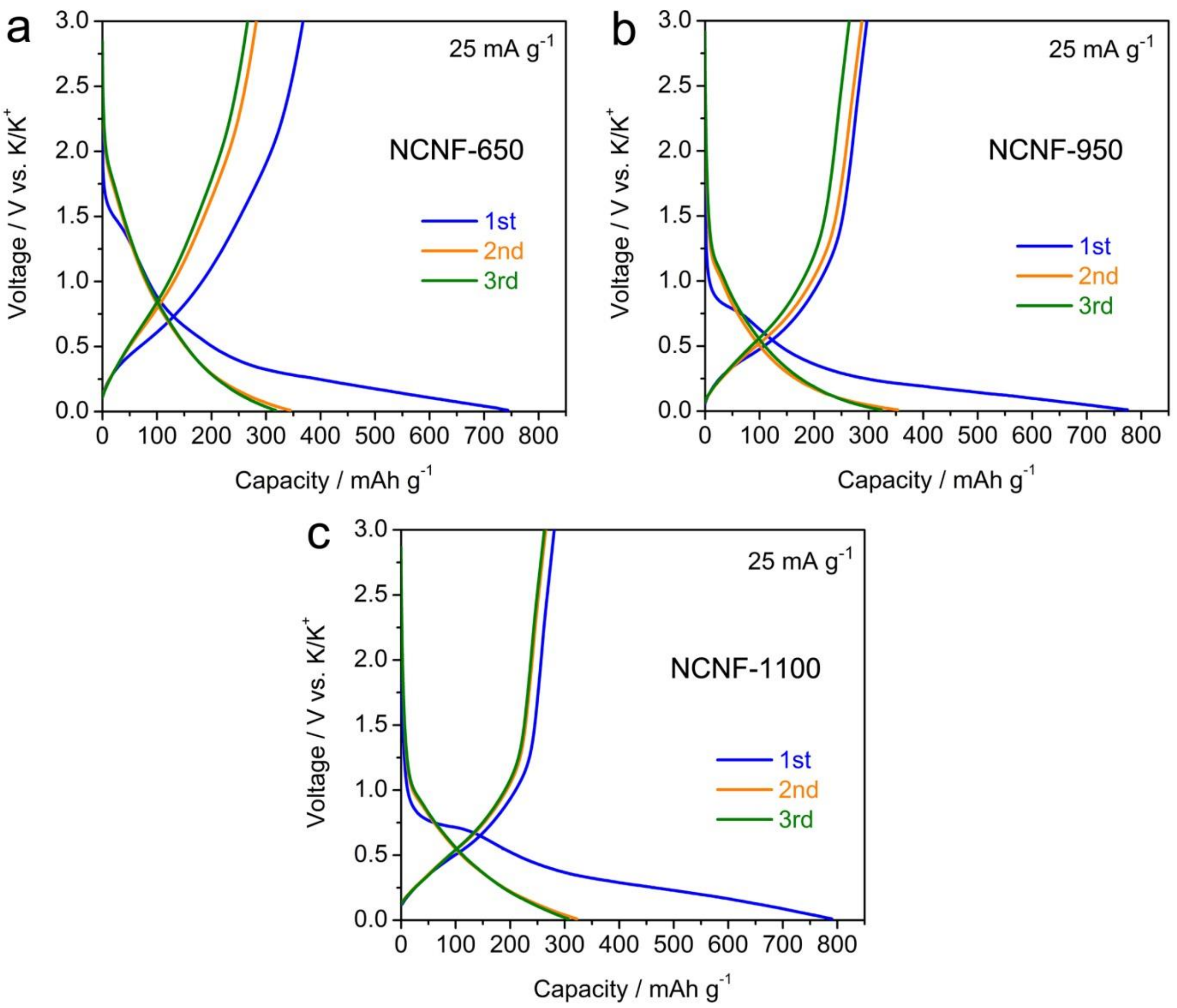

Supplementary Figure 8 | Electrochemical performance of NCNFs in the first three cycles in half cells. Galvanostatic charge/discharge profiles of NCNF-650 (a), NCNF-950 (b), and NCNF-1100 (c) for the first three cycles.

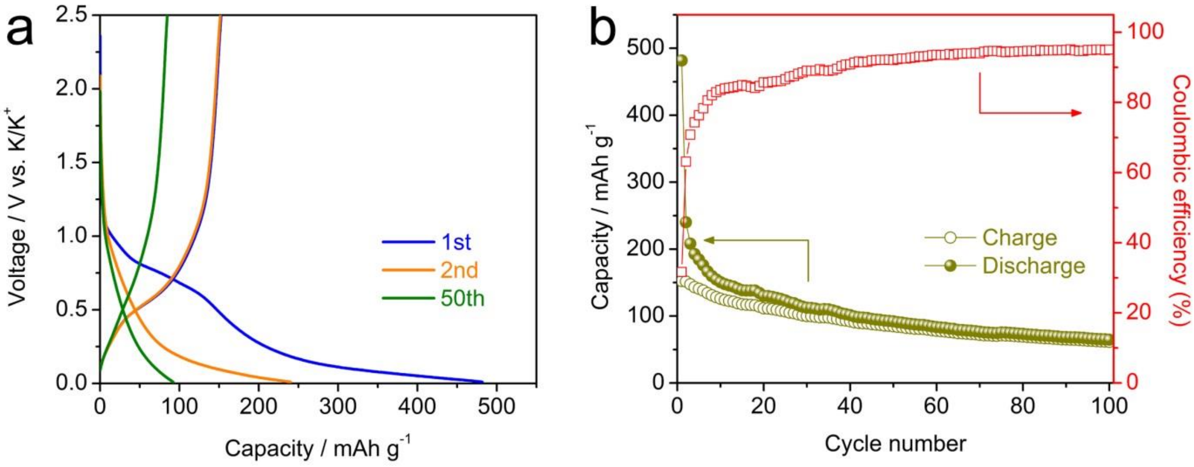

Supplementary Figure 9 | Electrochemical performance of Super P in a half cell. Galvanostatic charge/discharge profiles (a) and cycling performance (b) of the Super P half-cell at current density 50 mA g^{-1}.

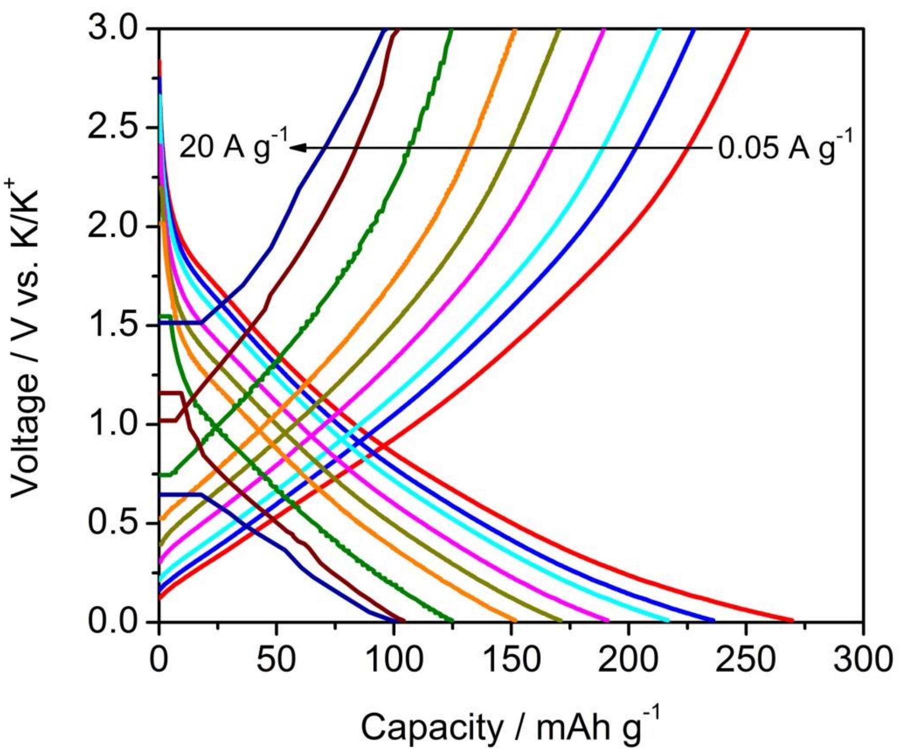

Supplementary Figure 10 | Rate capability of NCNF-650. Galvanostatic charge/discharge profiles of NCNF-650 at various current densities.

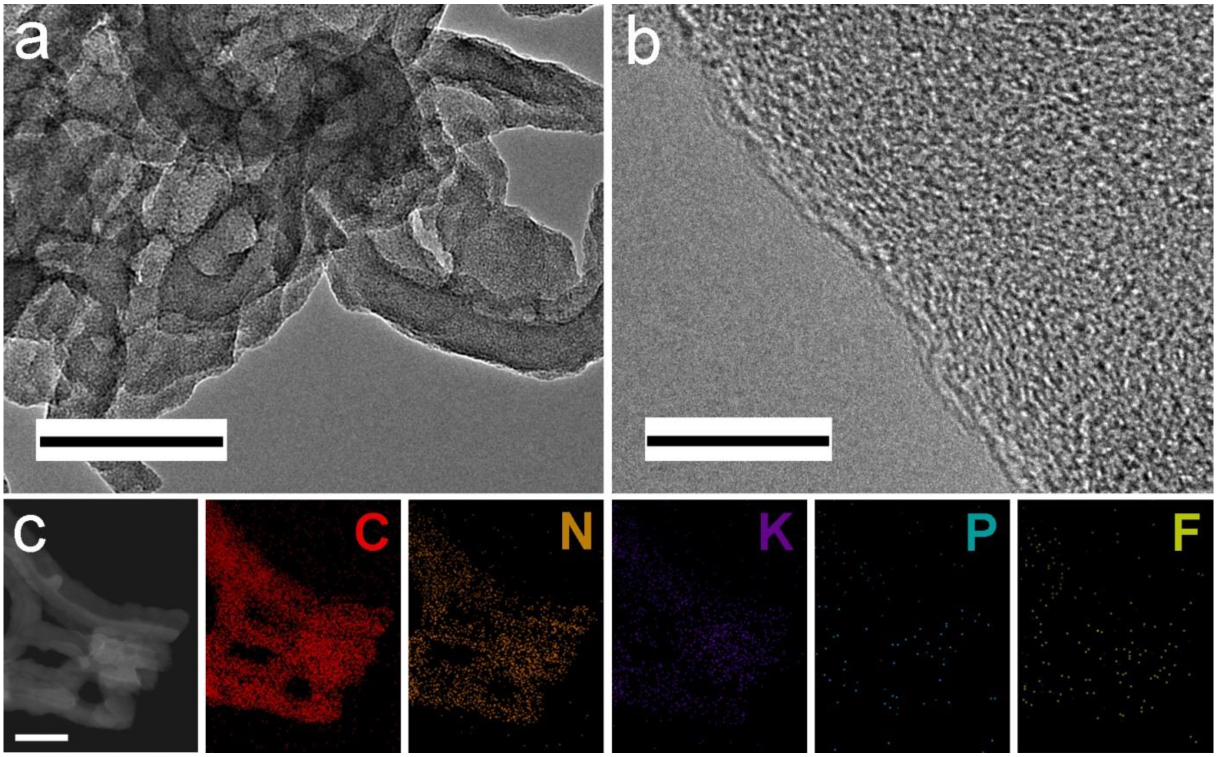

Supplementary Figure 11 | Post–cycling characterization of NCNF-650. Characterization of the NCNF-650 electrode after 4000 cycles: (a) TEM image; (b) HRTEM image; (c) images of element mapping. Scale bars: 100 nm (a and c); 10 nm (b).

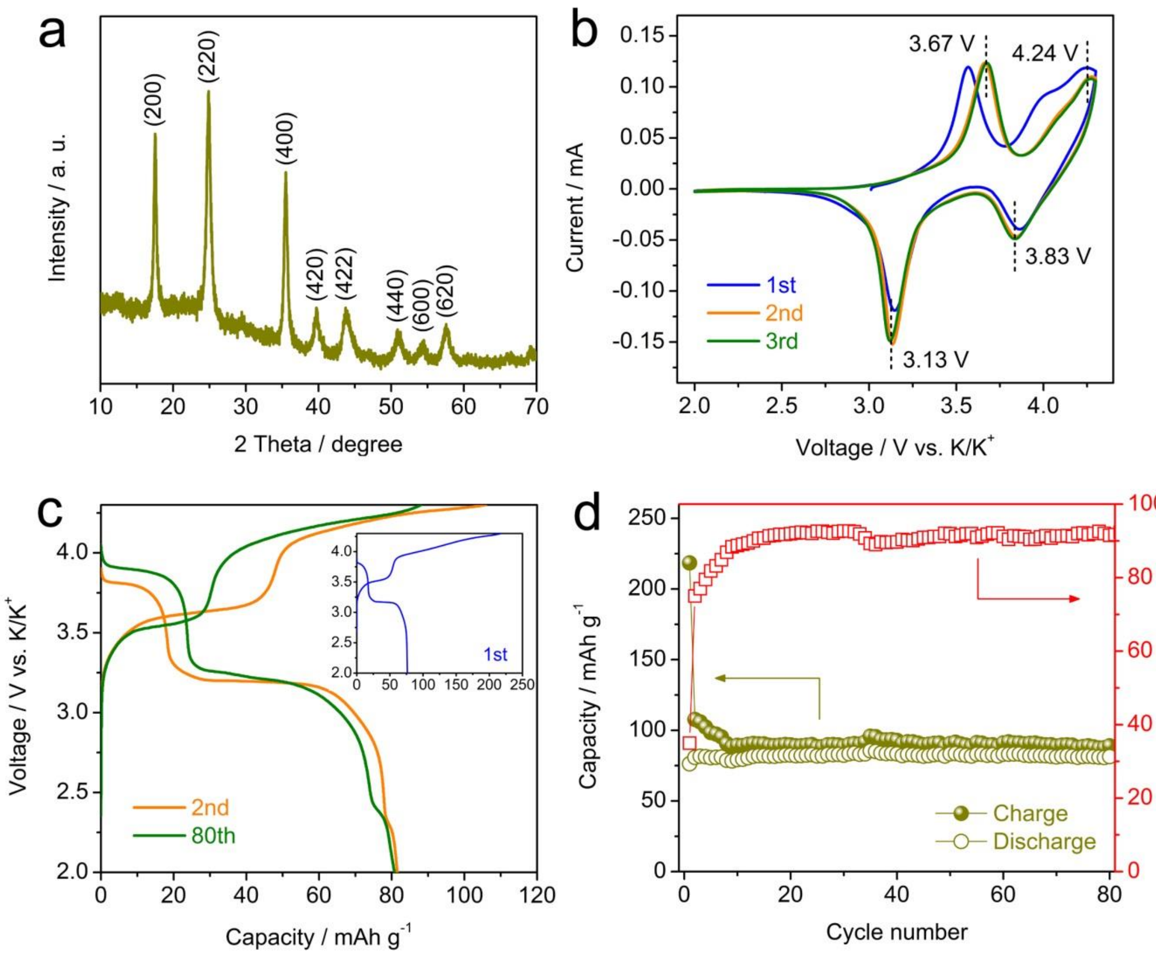

Supplementary Figure 12 | Characterization and electrochemical performance of KPB in a half cell. Characterization and electrochemical performance of KPB: (a) XRD pattern; (b) CV curves tested at scan rate of 0.1 mV s^{-1} and (c) galvanostatic charge/discharge profiles at 25 mA g^{-1} in a voltage window of 0.01–3.0 V (inset of (c): charge/discharge profile of the first cycle); (d) cycling performance at 50 mA g^{-1}.

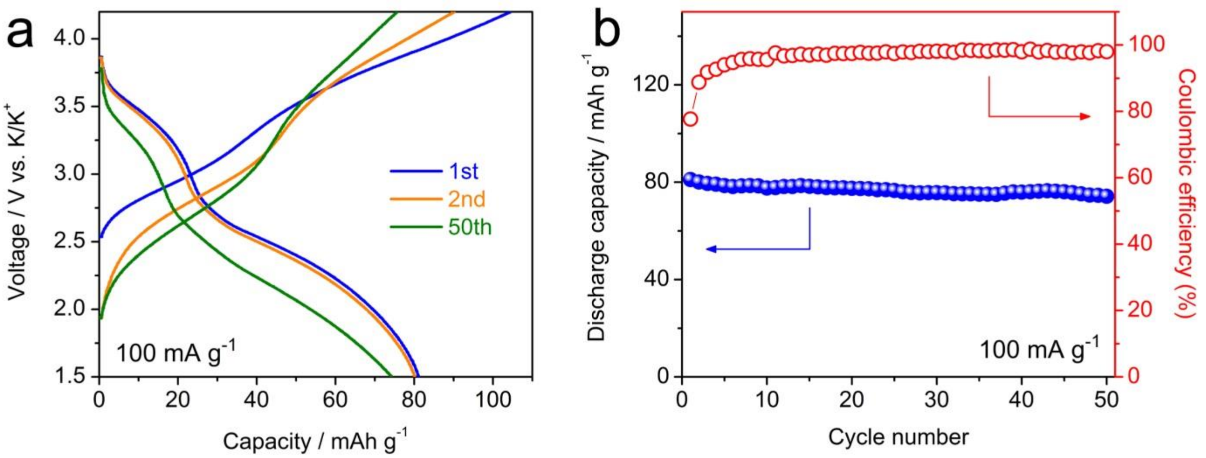

Supplementary Figure 13 | Electrochemical performance of the NCNF-650/KPB full cell. Electrochemical performance of the full-cell in the cathode-limited configuration: (a) galvanostatic charge/discharge profiles; (b) cycling performance at current density of 100 mA g^{-1}.

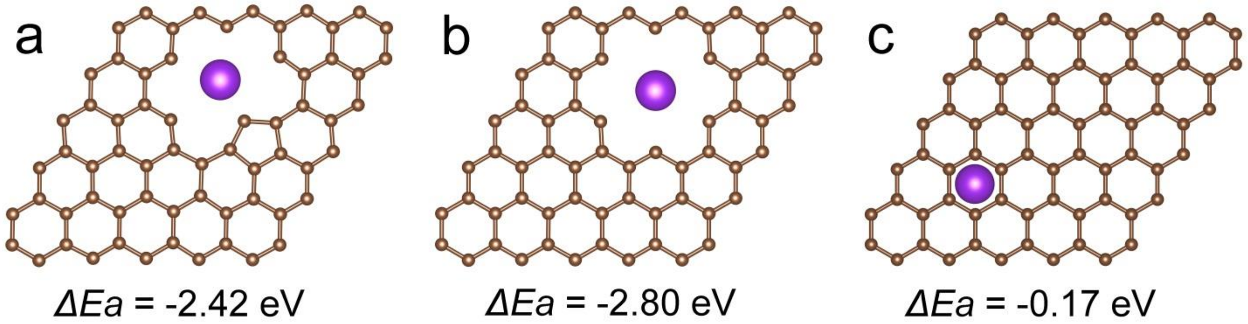

Supplementary Figure 14 | Theoretical simulations of defect carbon structures. Top view of a single K atom adsorbed in the C-5 (a), C-6 (b), and P-C (c) structures and the corresponding adsorption energy.

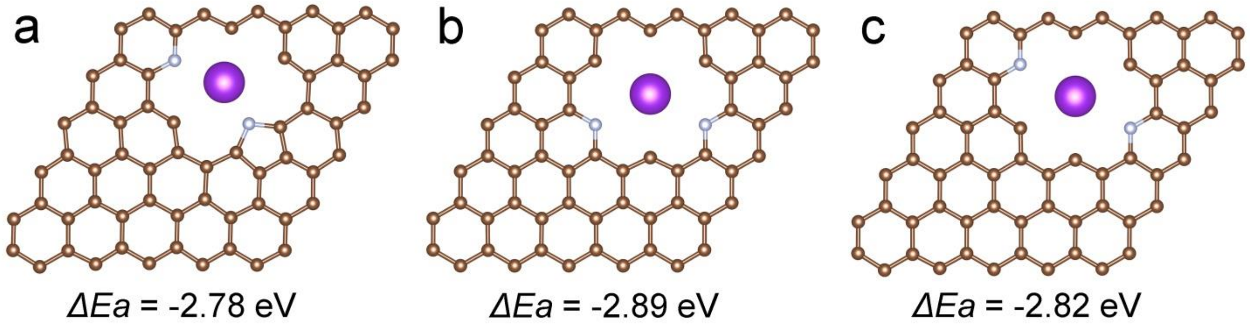

Supplementary Figure 15 | Theoretical simulations of nitrogen doped carbon structures. Top view of a single K atom adsorbed in the doped structures with two nitrogen atoms and the corresponding adsorption energy: (a) one N-5 and one N-6 atoms in the "meta-oriented" positions; (b) two N-6 atoms in the "meta–oriented" positions; (c) two N-6 atoms in the "para-oriented" positions.

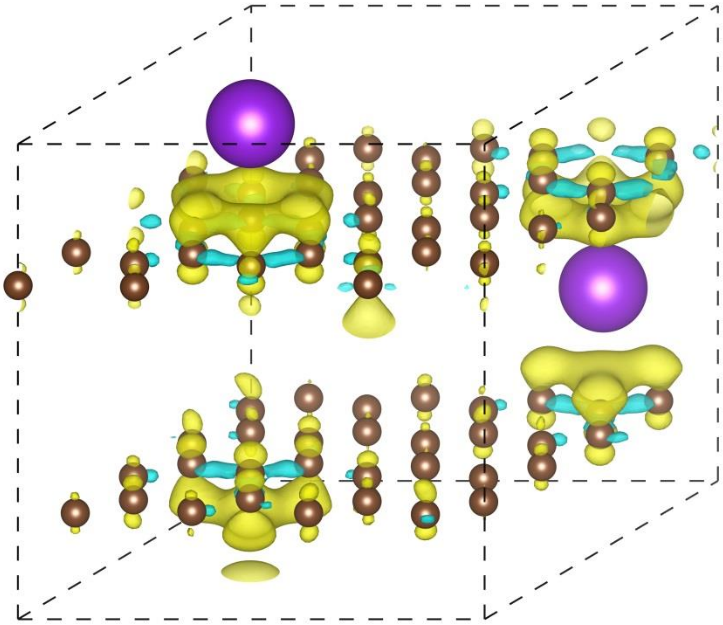

Supplementary Figure 16 | Electron density differences of K absorbed in the P-C structure. Yellow and blue areas represent increased and decreased electron density, respectively. The isosurfaces are the 0.0015 electron bohr^3. The brown, silver, and purple balls represent carbon, nitrogen, and potassium atoms, respectively.

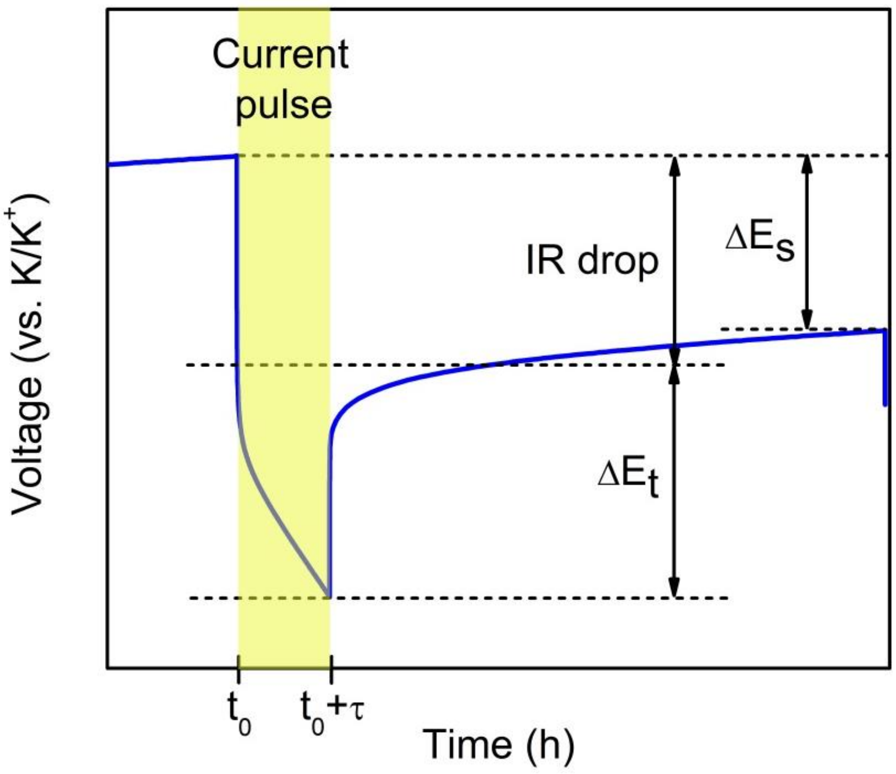

Supplementary Figure 17 | Calculation of diffusion coefficient. Schematic of the calculation of diffusion coefficient using the GITT technique.

Supplementary Notes

Supplementary Note 1 | The empirical *R* value is determined by the ratio of the intensity of the (002) peak height (A) to the background at the equivalent peak height (B)[1], as shown in Supplementary Figure 4. A value of 1.0 indicates a fully disordered structure while a value larger than 1.0 suggests a progressively increased degree of graphitization. The *R* values are 2.5, 3.6, and 4.3 for NCNF-650, NCNF-950, and NCNF-1100, respectively. It indicates the nature of soft carbon for the NCNFs and a higher degree of graphitization at higher carbonization temperature.

Supplementary Note 2 | The spectrum in Supplementary Figure 5 can be deconvoluted into two contributions centered at 399.8 (N-5) and 400.7 eV (N^+-5), both of which correspond to the N atoms within the pentagonal pyrrole rings. The N^+-5 contribution is associated with the benzenoid amine (–NH–) and protonation benzenoid amine (–NH^+–) within the rings due to the p–doped state of the as-prepared PPy. Same results were observed in other works that used oxidative polymerization of pyrrole[2,3].

Supplementary Note 3 | The spectrum in Supplementary Figure 6 can be deconvoluted into four contributions centered at 284.8, 285.9, 287.7, and 291.3 eV, corresponding to the graphitic carbon (C–C), the carbon in the C–N and C–O bonds, the carbon bound to one oxygen atom (C=O), and the carbon bound to two oxygen atoms (O=C–O), respectively[4,5]. The first two are the primary C groups. By increasing temperature, the amount of the graphitic carbon relative to the carbon in the C–N increases, suggesting the reduced N content and the increased degree of graphitization.

Supplementary Note 4 | Super P delivers the first charge and discharge capacities of 153 and 482 mAh g^{-1} with the first CE of 32% (Supplementary Figure 9a). It retains a discharge capacity of 65 mAh g^{-1} after 100 cycles, being 27% of the second discharge capacity (Supplementary Figure 9b). Both reversible discharge capacity and cyclability are significantly lower than the NCNFs, and as a result, its contribution to the overall capacity of the electrodes can be expected to be very small.

Supplementary Note 5 | The XRD pattern (Supplementary Figure 12a) is almost identical to that of $Fe_4[Fe(CN)_6]_3$ (JCPDS No. 52–1907) and in an agreement with the pattern in our previous

work[6], suggesting the successful synthesis of KPB. Two pairs of peaks (3.83/4.24 V and 3.13/3.67 V) are identified in the CV curves (Supplementary Figure 12b), being in accordance with the reported KPB based PIB cathodes[7]. Charge/discharge profiles shown in Supplementary Figure 12c display two defined discharge/charge plateaus that correspond to the observed CV peaks. The KPB cathode delivers the first charge/discharge capacities of 218/76 mAh g^{-1} with the first CE of 35%. It shows a good cyclability with almost no discharge capacity decay after 80 cycles (Supplementary Figure 12d).

Supplementary Note 6 | The diffusion coefficient is calculated according to Supplementary Equation (1) if the cell potential is linearly proportional to $\tau^{1/2}$:[8,9]

$$D = \frac{4}{\pi\tau}\left(\frac{m_b V_M}{M_B S}\right)^2\left(\frac{\Delta E_s}{\Delta E_t}\right)^2 \quad \left(t \ll \frac{L^2}{D}\right) \qquad (1)$$

where τ is the current pulse time (s), m_b is the mass of the active material, M_B is the molar mass of the active material, V_M is the molar volume of the active material, S is the geometric area of the electrode, and ΔE_s and ΔE_t are defined as shown in Supplementary Figure 17.

Supplementary References

1. Liu, Y., Xue, J., Zheng, T. & Dahn, J. Mechanism of lithium insertion in hard carbons prepared by pyrolysis of epoxy resins. *Carbon* **34**, 193–200 (1996).
2. Zhu, J., Xu, Y., Wang, J., Wang, J., Bai, Y. & Du, X. Morphology controllable nano-sheet polypyrrole-graphene composites for high–rate supercapacitor. *Phys. Chem. Chem. Phys.* **17**, 19885–19894 (2015).
3. Zhu, J. et al. Porous and high electronic conductivity nitrogen-doped nano-sheet carbon derived from polypyrrole for high-power supercapacitors. *Carbon* **107**, 638–645 (2016).
4. Wang, S., Xia, L., Yu, L., Zhang, L., Wang, H. & Lou, X. W. D. Free-standing nitrogen-doped carbon nanofiber films: integrated electrodes for sodium-ion batteries with ultralong cycle life and superior rate capability. *Adv. Energy Mater.* **6**, 1502217 (2016).
5. Guan, Z., Liu, H., Xu, B., Hao, X., Wang, Z. & Chen, L. Gelatin-pyrolyzed mesoporous carbon as a high-performance sodium-storage material. *J. Mater. Chem. A* **3**, 7849–7854 (2015).
6. Zhang, C. et al. Potassium Prussian blue nanoparticles: a low-cost cathode material for potassium-ion batteries. *Adv. Funct. Mater.* **27**, 1604307 (2017).
7. Bie, X., Kubota, K., Hosaka, T., Chihara, K. & Komaba, S. A novel K-ion battery: hexacyanoferrate(II)/graphite cell. *J. Mater. Chem. A* **5**, 4325–4330 (2017).
8. Weppner, W. & Huggins, R. A. Electrochemical investigation of the chemical diffusion, partial ionic conductivities, and other kinetic parameters in Li_3Sb and Li_3Bi. *J. Solid State Chem.* **22**, 297–308 (1977).
9. Jian, Z., Xing, Z., Bommier, C., Li, Z. & Ji, X. Hard carbon microspheres: potassium-ion anode versus sodium–ion anode. *Adv. Energy Mater.* **6**, 1501874 (2016).
